# Varenicline and Bupropion for Long-Term Smoking Cessation (the MATCH Study): Protocol for a Real-World, Pragmatic, Randomized Controlled Trial

**DOI:** 10.2196/10826

**Published:** 2018-10-18

**Authors:** Laurie Zawertailo, Tara Mansoursadeghi-Gilan, Helena Zhang, Sarwar Hussain, Bernard Le Foll, Peter Selby

**Affiliations:** 1 Department of Pharmacology and Toxicology University of Toronto Toronto, ON Canada; 2 Nicotine Dependence Service Centre for Addiction and Mental Health Toronto, ON Canada; 3 Translational Addiction Research Laboratory Centre for Addiction and Mental Health Toronto, ON Canada; 4 Department of Family and Community Medicine Faculty of Medicine University of Toronto Toronto, ON Canada; 5 Department of Psychiatry University of Toronto Toronto, ON Canada; 6 Department of Institute of Medical Sciences University of Toronto Toronto, ON Canada; 7 Acute Care Program Centre for Addiction and Mental Health Toronto, ON Canada; 8 Campbell Family Mental Health Research Institute Centre for Addiction and Mental Health Toronto, ON Canada; 9 Dalla Lana School of Public Health University of Toronto Toronto, ON Canada

**Keywords:** bupropion, genetics, internet, personality traits, smoking cessation, tobacco, varenicline

## Abstract

**Background:**

Varenicline and bupropion are efficacious, prescription-only pharmacotherapies for smoking cessation; however, their real-world impact is limited by prescriber knowledge, affordability, and accessibility.

**Objective:**

The primary objective of the MATCH (Medication Aids for Tobacco Cessation Health) study was to evaluate the real-world, long-term effectiveness of mailed bupropion and varenicline in a sample of interested smokers with the utilization of Web-based recruitment and follow-up. In addition, the study aims to investigate the genotypic and phenotypic predictors of cessation.

**Methods:**

This is a two-group, parallel block, randomized (1:1) open-label clinical trial. This study will be conducted online with the baseline enrollment through the study’s website and follow-up by emails. In addition, medication prescriptions will be filled by the study contract pharmacy and couriered to participants. Individuals who smoke ≥10 cigarettes per day and intend to quit within the next 30 days will be recruited through Public Health Units and Tobacco Control Area Networks throughout Ontario by word-of-mouth and the internet. Eligible participants will receive an email with a prescription for 12-week assigned medication and a letter to take to their physician. The recruitment and randomization will continue until 500 participants per arm have received medication. All participants will receive weekly motivational emails during the treatment phase. The primary outcome measure is the smoking status after 6 months, biochemically confirmed by mailed-in salivary cotinine. Follow-ups will be conducted through emails after 4, 8, 12, 26, and 52 weeks of starting the treatment to assess the smoking prevalence and continuous smoking abstinence. In addition, mailed-in saliva samples will be used for genetic and nicotine metabolism analyses. Furthermore, personality characteristics will be assessed using the Big Five Aspect Scales.

**Results:**

The project was funded in 2014 and enrollment was completed in January 2017. Data analysis is currently underway.

**Conclusions:**

To the best of our knowledge, this is the first randomized controlled trial to mass distribute prescription medications for smoking cessation. We expect this method to be logistically feasible and cost effective with quit outcomes that are comparable to published clinical trials.

**Trial Registration:**

ClinicalTrials.gov NCT02146911; https://clinicaltrials.gov/ct2/show/NCT02146911 (Archived by WebCite at http://www.webcitation.org/72CZ6AvXZ)

**Registered Report Identifier:**

RR1-10.2196/10826

## Introduction

### Background

The prevalence of tobacco smoking in developed countries has steadily declined over the past three decades. However, with 16% of the general Canadian population aged ≥15 years [1], the prevalence of smoking remains a formidable cause of mortality and morbidity in Canada. The burden of tobacco use is high, resulting in Can $17 billion in direct and indirect costs to the Canadian economy [2]. Clinical interventions are an important component of comprehensive tobacco control strategies [3]. Nicotine replacement therapy, bupropion, and varenicline are proven to be efficacious pharmacological aids, doubling the chances of success in quitting smoking [4-6].

To date, 3 randomized controlled trials (RCTs) have compared varenicline, bupropion, and placebo [6-8]. The primary outcome measure was continuous abstinence rates (CARs) at various time points as follows: weeks 9-12 (end of treatment), weeks 9-24 (6-month time point), and weeks 9-52 (1-year time point). Only one trial reported a significant difference in CARs after 52 weeks [7] with varenicline having significantly higher long-term abstinence rates than bupropion (23% vs 14.6%, respectively; odds ratio [OR] 1.77, 95% CI 1.19-2.63, *P*=.004). However, an identical clinical trial conducted in parallel with the same sample size [6] did not show a significant difference in CARs between varenicline (21.9%) and bupropion (16.1%; OR 1.46, 95% CI 0.99-2.17, *P*=.06) after 52 weeks.

Therefore, although there is limited evidence for the superior long-term efficacy of varenicline, no study has assessed the real-world effectiveness of these medications for long-term abstinence. This is important because of some significant differences between clinical trials and real-world settings, which could influence cessation treatment outcomes; for example, clinical trials have strict eligibility criteria, excluding participants with certain comorbidities. Therefore, participants in these studies are in better health compared with the general population. In addition, treatment with medications in clinical trials is accompanied by one-on-one smoking cessation behavioral counseling often on a weekly basis, which is largely unavailable in real-world settings [7]. These factors together have the potential to restrict the external validity of clinical trial findings. As such, there is a need to assess the real-world effectiveness of these prescription medications at a population level to further strengthen the evidence base for the effective treatment of tobacco dependence.

Even though proven efficacious in clinical trials, according to the Canadian Tobacco Use Monitoring Survey in 2007, less than half of smokers who have ever attempted to quit have used a smoking cessation aid [9]. Most of these smokers identify the lack of access to adequate and evidence-based information, in addition to the cost, as reasons for not using these smoking cessation pharmacotherapies [9]. Furthermore, bupropion and varenicline are only available by a prescription from a licensed practitioner. Therefore, in addition to knowledge and affordability, their population-level impact is limited by accessibility. Furthermore, smoking cessation clinics are limited in number, and a survey conducted in Canada demonstrated that the occurrence of smoking cessation discussion between physicians and patients is not common. In fact, of 88% of smokers who visited a primary care physician in the year prior, only half received any advice on quitting or reducing smoking [10]. Efforts to address these barriers could greatly improve the use and effectiveness of these smoking cessation medications in real-world settings [11]. Furthermore, mass distribution approaches, bypassing clinics and physicians, have been successful for nicotine replacement therapy [12,13]. However, bupropion and varenicline have the potential to make a greater impact, given their superior results from clinical trials.

### Study Aims

The primary aim of this large RCT is to assess the long-term cessation rates associated with bupropion and varenicline treatment using the internet as a novel approach. Secondary aims include investigating the pharmacogenetic factors and phenotypic characteristics affecting nicotine dependence and smoking cessation outcomes.

### Hypotheses

The hyphotheses of the study are as follows: the long-term abstinence rates (at 6-month follow-up) will be significantly higher in the varenicline group than in the bupropion group; the overall quit rates will be similar to those reported from traditional RCTs; and specific genotypes and phenotypes of individuals will influence smoking treatment outcomes.

## Methods

### Study Design

**Figure 1:**
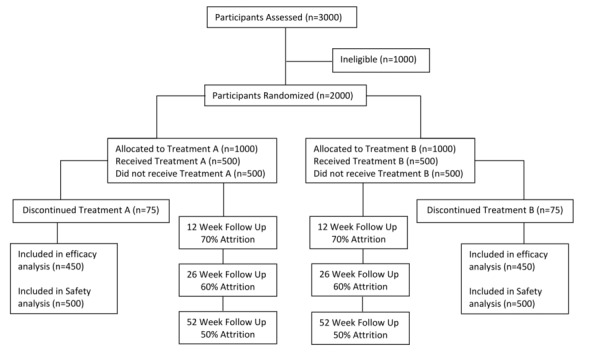
An overview of the proposed intervention trial (CONSORT [Consolidated Standards of Reporting Trials] diagram).

The Medication Aids for Tobacco Cessation Health (MATCH) study is an internet-based pragmatic randomized clinical trial. This study is open-label, wherein eligible participants are randomly assigned to study medication, bupropion (Zyban) or varenicline (Champix), for 12 weeks in conjunction with weekly motivational emails. All participants will receive medication plus an identical email-based behavioral intervention. The research methods and protocol for this study have been approved by the standing Research Ethics Board (REB) at the Centre for Addiction and Mental Health (CAMH) with the reference number 200/2012. This study is registered at ClinicalTrials.gov (NCT02146911). Figure 1 shows a CONSORT (Consolidated Standards of Reporting Trials) diagram for the study outlining estimates for the recruitment and dropout. The figure illustrates the estimated participant flow of the proposed intervention trial. It demonstrates the expected participant numbers in the enrollment and study completion processes. The estimated number of participants active at each stage of the study are estimated based on the pilot study conducted previously [14].

### Setting

#### Web-Based Consent, Self-Assessment, and Automated Eligibility Determination

**Figure 2:**
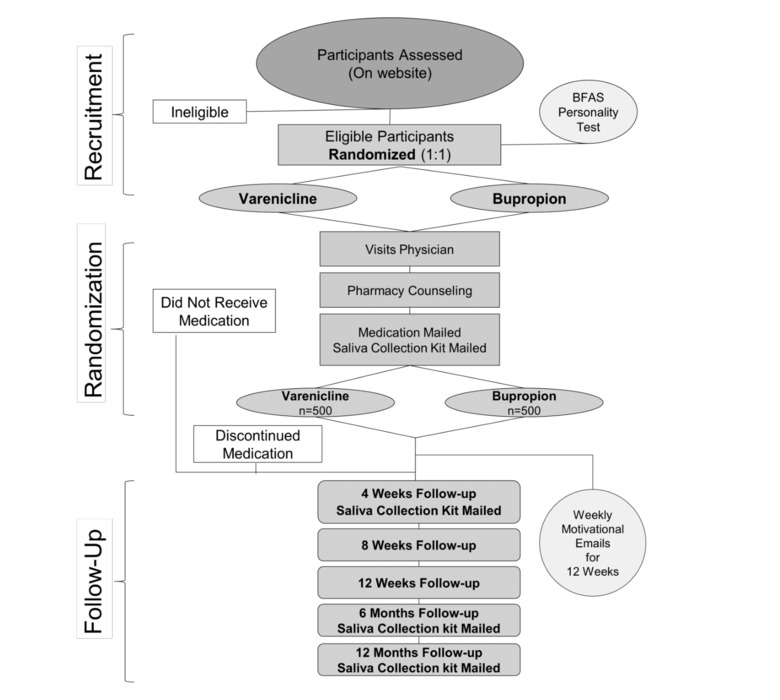
Participants’ flowchart. BFAS: Big Five Aspect Scale.

Figure 2 shows the flowchart of participants through different components of the study. It includes the enrollment process, followed by randomization, mailing of the medication, and follow-up surveys completed at various time points.

Interested individuals will visit the study website and read a brief description of the study purpose, its procedures, and treatments provided. Those interested in participating will read the study information and consent form and provide their consent by clicking “yes” on the study website; they may also indicate whether they would like to be contacted for future studies. Individuals are permitted to participate in the study even if they do not give consent to be contacted for future studies. After completing the consent form, participants will complete the baseline survey, which will take approximately 20 minutes. The baseline questionnaire collects information on demographics, socioeconomic factors, education, ethnicity, smoking habits (cigarettes smoked per day, duration of daily smoking, and when participants first began smoking), importance and confidence to quit, substance and alcohol usage, concurrent usage of other medication(s), and any psychiatric comorbidities (ie, depression, anxiety, and schizophrenia).

In addition, if eligible, participants will be asked if they consent to provide a saliva sample for the genetic testing component of the study. If they agree to enroll in this substudy, they will complete the Big Five Aspect Scale personality test online through the study website, which will take approximately 10-15 minutes. Big Five Aspect Scale is a self-reported public domain test, which assesses the big 5 personality traits by asking 100 questions answered on a 5-point scale ranging from “strongly disagree” to “strongly agree” [14]. Individuals will be permitted to take part in the efficacy study even if they do not consent to participation in the genetics substudy. The questionnaires used have been published previously and include the Fagerstrom test for nicotine dependence [15], the BFAs for accessing the big 5 personality traits—extraversion, neuroticism, conscientiousness, agreeableness, and openness to experience [14]—and Patient Health Questionnaire for the evaluation of depression [16]. These questionnaires have been widely used in clinical studies, involving smoking, and extensively validated [14-16].

After submitting the completed questionnaires, individuals will be notified, through an on-screen message, whether they are eligible for the study. Those who are eligible will receive an email, including their medication assignment, the study information and consent form, a standardized letter to the doctor to convey information about the study, and a standard script for the study medication they have been assigned to for their physician (or other licensed prescriber) to sign. Because there are no in-person study visits, Salivette kits will be mailed out at the time of study enrollment to verify the participants’ smoking status.

In addition, baseline saliva sample will be used to measure the nicotine metabolite ratio, which is the ratio of 3'-hydroxycotinine and cotinine (COT) concentration; this is a validated phenotypic biomarker of the CYP2A6 enzyme activity [17]. Furthermore, Salivette kits will be sent at weeks 4, 26, and 52 to measure the salivary COT level for the biochemical verification of abstinence. Those who consent to the genetics substudy will receive a second email with the genetics study information and consent form and the Oragene saliva DNA kits by mail to collect a DNA sample for the pharmacogenetics component of the study. Participants will be compensated for each DNA (if applicable) and subsequent saliva samples received.

#### Patient Visit to a Licensed Practitioner

All eligible participants will be required to visit their physician or another licensed prescriber within 5 weeks from the enrollment date to sign the prescription for their assigned medication. Reminder emails will be sent after 2 weeks of the enrollment date to make sure that the participants have booked an appointment to visit their practitioner. At the visit, participants will discuss their medical history, the medications they are on, and any other concerns they have regarding the treatment. At his or her discretion, a licensed practitioner may either sign the prescription for study medication or choose not to prescribe the assigned medication to their patient. The practitioner’s office will then fax the signed prescription to a mail-order pharmacy contracted to do the study that will then fill the prescription.

#### Mail-Out Pharmacy

Prescriptions signed by a licensed practitioner and verified to be authentic will be filled and mailed to the participants’ addresses by the contract mail-order pharmacy. In accordance with the Ontario College of Pharmacists’ standard of practice, pharmacists will call each study participant at the time of dispensing (ie, mailing) the medication to inform participants about directions to use the medication, discuss possible allergies, concomitant medications, and offer counseling.

### Participants

#### Inclusion and Exclusion Criteria

The following inclusion criteria will need to be met before any individual is randomly assigned to one of the 2 treatment arms: Ontario resident; having a valid email address; aged at least 19 years; current daily smoker; smoking at least 10 cigarettes per day; smoked daily for at least the past year; and have an intention to quit smoking within 30 days of receiving the medication. The following are criteria that will exclude an individual from being randomly assigned to one of the 2 treatment arms: a history of psychotic disorder (schizophrenia or bipolar disorder) or eating disorder; brain injury; seizure disorder; pregnancy, lactation, or at risk of becoming pregnant; allergy or sensitivity to bupropion or varenicline; or currently taking varenicline or Champix, bupropion or Zyban or Wellbutrin, monoamine oxidase inhibitors, thioridazine, antidepressants, or other medications containing bupropion hydrochloride.

#### Recruitment and Randomization

The primary method of recruitment for this study will be by word-of-mouth through family, friends, or health care providers. The Smoking Treatment for Ontario Patients Study [12,13], also conducted by the investigators of this trial, has successfully built a collaborative network of providers from public health units, community health centers, family health teams, and community pharmacies across Ontario. This network of providers has proven to be effective in disseminating new treatment opportunities to smokers in their area. Interested individuals will self-identify and will be directed to enroll through the study website. Moreover, as part of the consenting procedure for those who participated in the Smoking Treatment for Ontario Patients Program, individuals will be asked whether they would like to be contacted about new research opportunities by CAMH. Those who choose “yes” will then be included in a database forming a smokers’ registry that can be contacted in case new research opportunities come up. This smokers’ registry will be another method of recruitment used for this study. Furthermore, the public will be informed about the study through CAMH social media accounts, Smokers’ Helpline, and Facebook advertisements.

#### Randomization

Eligible participants will be randomly assigned to one of the 2 study arms (varenicline or bupropion). Permuted block randomization in a 1:1 ratio in blocks of 100 will be employed. The randomization process will be computerized. Owing to the large proposed sample size in this study, there will be no stratification or minimization.

#### Current Recruitment Status

Study recruitment is no longer active; however, there are still few 1-year follow-ups that are yet to be completed by participants.

#### Retention Strategies

Weekly motivational emails and payment for mailed-in saliva samples at the 4-week time point will be used to encourage treatment fidelity and maintain engagement in the study.

#### Sample Size

In the 2 head-to-head RCTs of varenicline versus bupropion [6,7], participants were randomized 1:1:1 to varenicline, bupropion, or placebo arms with 341-345 participants in each group. In the trial conducted by Gonzales et al [6], the 9- to 52-week CARs were 21.9% in the varenicline group versus 16.1% in the bupropion group (OR 1.49, 95% CI 0.99-2.17, *P*=.057). In the trial conducted by Jorenby et al [7], the 9- to 52-week CARs were 23% in the varenicline group compared with 14.6% in the bupropion group (OR 1.77, 95% CI 1.19-2.63; *P*=.004). Because this is an effectiveness trial, we anticipate lower quit rates overall. Indeed, in our previous feasibility study [18], 7-day point prevalence abstinence (PPA) rates after 6 months (intention-to-treat analysis) were 18.9% in those who received varenicline and 17.4% in those who received bupropion.

To be sufficiently powered to detect a significant difference in the 26-week CARs, we plan to randomize 500 subjects to each medication arm. We predict that the real-world abstinence rates will be slightly lower than those observed in clinical trials and may be similar to what we found in our nonrandomized study that this protocol is based on [19]. Therefore, we assumed that the 26-week CAR in the varenicline-treated group would be 20% compared with 15% in the bupropion-treated group. At 500 subjects per group, we will have 80% power to detect a significant difference between groups at a .05 level of significance.

#### Blinding

This is an open-label, clinical trial; therefore, the procedures will not be blinded because participants will need to visit their physician to approve their randomized medication. Pharmacists, primary care providers and research personnel will know which medication participants have been assigned to.

### Medication

Compliance with the prescribed regimen will be measured by participants’ self-report follow-up assessments at weeks 4, 8, and 12. In addition, saliva sample kits will be mailed out to participants at week 4 for the biochemical analysis of drug levels to confirm medication compliance. Participants will be compensated for each sample received.

#### Behavioral Support

All eligible participants will receive 12 weekly motivational emails for the duration of treatment. The emails include tips on several things, other than the medications, which participants can do to help them quit smoking. The contents of the emails vary from week to week (Multimedia Appendix 1).

#### Participant Follow-Up by Email

Participants will be contacted by email at weeks 4, 8, 12, 26, and 52 to complete follow-up surveys. The follow-up surveys will collect data on changes to the participants’ smoking pattern and medication use and any possible adverse reactions experienced. During each follow-up survey, participants will be asked whether they have experienced any adverse events. If the participants at any point are experiencing intolerable adverse events, they can contact the study personnel to withdraw from the study. They will be compensated for all the saliva and genetic samples that they had submitted prior to being withdrawn. There will be no modifications to interventions even if adverse side effects are reported. Instead, participants will either be discontinuing their treatment or will be advised to speak with their physician regarding the intervention. If participants withdraw from the study, they will still be asked to complete follow-up surveys.

#### Cost-Effectiveness

To make an impact on the prevalence of smoking in the overall population, it is necessary for an intervention to reach a high number of people. Efficacious pharmacotherapies are available for smoking cessation but their reach is typically limited. To the best of our knowledge, this is the first study to attempt the mass distribution of prescription medications for smoking cessation using a randomized study design. The method proposed has been previously demonstrated by the investigators of this study to be logistically feasible and effective in terms of cessation rates [18] and has provided crucial evidence for an approach that has the potential to make a significant impact on cessation rates at a population level by demonstrating a way to take full advantage of the available smoking cessation aids.

All recruitment, consenting, and data collection are Web-based. The mail-order pharmacy is less expensive than dispensing through a research pharmacy, and we do not need to pay physicians to recruit their patients into studies because subjects self-identify first and then go to their physician (in Canada, all physician visits are paid for by our universal health care system). As such, this large randomized trial can be extremely cost effective with an estimated cost of under Can $150,000. We aim to provide medication to 1000 participants; therefore, the cost per enrollment is estimated to be Can $133.33. The 52-week CAR in the varenicline-treated group is assumed to be 18% compared with 12.5% in the bupropion group. Therefore, the cost per quit is estimated to be about Can $873.36 plus Can $33.70 for a visit to the prescriber if the government’s universal health plan is billed; this is much lower compared with the economic burden of continued smoking. A report published in 2015 estimated the annual health care cost in Canada to be Can $3071 per smoker [20].

### Primary Outcome

The primary outcome measures will be related to the effectiveness of treatment. The primary outcome will biochemically confirm 30-day continuous abstinence by mailed-in salivary COT 26 weeks after the start of treatment. Secondary outcome variables will be self-reported 30-day continuous abstinence at the end of the treatment (weeks 9-12) and at weeks 26 and 52; this is defined as not having smoked, even a puff, in the past 30 days and lack of relapse during this period. Other outcome variables include self-reported 7-day PPA measured at weeks 4, 8, 12, 26, and 52. The 7-day PPA is defined as not having smoked, even a puff, over the last 7 days.

### Exploratory Measures and Potential Covariates

The tertiary outcome measures will be related to variations in genetic polymorphisms, metabolic factors, and personality traits observed in each treatment group. A 3-way analysis looking at the interaction between genetic polymorphism and treatment outcome, personality traits and treatment outcome, and genetic polymorphism and personality traits will be conducted; this part of the study should be considered exploratory. We have no specific hypotheses currently.

### Data Access

Datasets will only be available to CAMH research personnel only or those involved in the study. The funder does not have access to the trial dataset. Currently, there is no ability for the study data to be openly accessed by other researchers because of the restrictions placed by our REB.

### Data Analysis

Analysis of study results will be conducted using the intention-to-treat analysis, wherein all participants who are randomized to a treatment arm and receive their assigned medication are included in the final analysis whether they complete the study or respond to follow-up surveys at study end points. This method will be used to avoid any bias that can potentially arise because of crossover and dropouts, affecting the initial random assignment to treatment groups. Participants who are lost to follow-up or do not provide a saliva sample for the COT confirmation of abstinence will be considered as smokers according to recommendations by the Society for Research on Nicotine and Tobacco’s subcommittee on Biochemical Verification (2002) [21].

The baseline characteristics will be analyzed and compared between intervention groups using the Student’s *t* test for continuous variables and cross-tabs chi-square analysis for all categorical variables. Any characteristics that differ significantly across our 2 study groups will be included as covariates in all subsequent analyses. The effect of intervention over time will be evaluated using the longitudinal logistic regression analysis with abstinence as the dependent variable and treatment condition along with other baseline characteristics as independent predictors. We will construct a longitudinal generalized estimating equations (GEE) model for each binary outcome. GEE is a suitable analysis method because it accommodates for the statistical dependence among repeated observations within subjects. Because our primary outcome is abstinence from smoking (Yes or No) at each follow-up time point, GEE is appropriate because it allows for the estimation of population-averaged effects while accounting for the dependencies among the repeated measures. The data analysis for this study will be overseen by the principal investigator in consultation with the Biostatistical Consulting Service at CAMH.

## Results

The project was funded in 2014 and enrollment was completed in January 2017. Data analysis is currently underway and the first results are expected to be submitted for publication in October 2018.

## Discussion

The proposed trial on providing free medication mailed to smokers is expected to be cost effective and will be useful for policy makers to consider as part of a comprehensive tobacco control strategy. This can help reduce the prevalence of smoking and its related costs to our health care system and the overall economy. With the knowledge gained, the method can be modified according to the peculiarities of other health care jurisdictions to impact the smoking prevalence in those areas as well.

## Appendix

Multimedia Appendix 1 Example emails of MATCH weekly motivational messages.

## Abbreviations

CAMH: Centre for Addiction and Mental Health
CAR: continuous abstinence rate
CONSORT: Consolidated Standards of Reporting Trials
COT: cotinine
GEE: generalized estimating equation
MATCH: Medication Aids for Tobacco Cessation Health
OR: odds ratio
PPA: point prevalence abstinence
RCT: randomized controlled trial
REB: Research Ethics Board
