# Supplementary material for: Varenicline and Bupropion for Long-Term Smoking Cessation (the MATCH Study): Protocol for a Real-World, Pragmatic, Randomized Controlled Trial
Source: JMIR Res Protoc. 2018 Oct 18;7(10):e10826. doi: 10.2196/10826 (PMC6231835; doi:10.2196/10826)
Supplement: Multimedia Appendix 1 [file resprot_v7i10e10826_app1.pdf]

Appendix I. Email example of MATCH weekly motivational messages.

MATCH Weekly Motivational Email Messages

Weekly Tip #1:

Creating a smoke-free environment is important during your quit attempt. Make a decision *not* to smoke in your home and vehicle and ask others to do the same. If your entire home cannot go smoke-free, explore areas where you can restrict smoking. At work, avoid smoking areas during your breaks. Making your physical environment smoke-free can help reinforce your decision to quit smoking.

Weekly Tip #2:

Support systems are important during any big change. Identify all of the positive supports in your life and tell them you are quitting smoking and need their support. Also identify any negative influences who may not want you to quit and figure out how you are going to deal with them during this time. Take advantage of other supports available to you, such as Smoker's Helpline, websites, your doctors or other health care providers. Surrounding yourself with positive and supportive people can help you quit and stay quit.

Weekly Tip #3:

Slips and lapses are a part of the quitting process and can be common. Use any slip or lapse as a learning experience. Identify what happened, how you could have prevented the situation, and what you can do if you're in the situation again. Use these experiences to re-assess your quit plan and then try quitting again. It is important that you realize your quit attempt is not over; refocus and restart immediately after your lapse. Remember, quitting smoking is a process not an event and may take several attempts before you get it right. If you're taking smoking cessation medications, it is very important that you continue taking the medication as directed.

Weekly Tip #4:

One of the benefits of quitting smoking is the amount of money you save. The price of a pack of cigarettes is about $12; so that means if you smoked about 15 cigarettes a day you would save about $810 in three months (enough to purchase a new 42-inch flat-screen LED HD TV) or $3,240 in one year (enough for a long vacation abroad or a whole new wardrobe). In 10 years you will have enough money to make a down payment on a house! Therefore, take advantage of quitting smoking and reward yourself. You deserve it and you can now afford it. You can also download a free quit meter by visiting http://www.dedicateddesigns.com/qk/. The quit meter with help you track various statistics and milestones as you quit smoking to keep you motivated.

Weekly Tip #5:

Quitting smoking is a significant change in your life that can transform how you think of yourself. Sit back and picture yourself as a confident non-smoker…close your eyes and visualize yourself socializing

with family and friends, going through your daily routines, or dealing with a problem. Imagine not having to think about smoking or searching for your cigarettes or matches. Now, feel yourself relaxed, see yourself confident and without the craving for a cigarette. Guess what? You'll be there sooner than you think!

Weekly Tip #6:

Your smoking may be associated with certain people, places, or things. These can act as triggers for you to want to smoke. Identify your personal triggers and think about how you will deal with them. For example, change your day-to-day routine or find alternative activities to smoking. Problem solving ahead of time can help you deal with these situations when they arise and help you quit and stay quit.

Weekly Tip #7:

While it's not easy for most people, quitting smoking has many positive results. In addition to the long-term health benefits of quitting smoking, there are many benefits you'll notice immediately. For example, within days and weeks of quitting smoking you may notice that you have more energy, better smell and taste, whiter teeth and fresher breath. To reinforce your motivation, make a list of all of the benefits of quitting smoking and keep it close by.

Weekly Tip #8:

There are many good reasons why people want to quit smoking. Sometimes it's easy to forget why you wanted to quit in the first place. Write down your personal reasons for quitting and use them as reminders when things seem tough. Your reasons may change over time so review your list regularly. Reminding yourself of all the reasons you want to quit can help you stay focused on achieving your goal.

Weekly Tip #9:

Quitting smoking can make a big difference to your health and the health of your family (and others who are around you). Among smokers who have already had a heart attack, quitting smoking reduces the chance of a second heart attack by 50%, compared to those who continue to smoke. Also, when non-smokers are exposed to second hand smoke, their risk of coronary heart disease is increased by more than 50%. The message is clear: when you quit smoking everyone benefits!

Weekly Tip #10:

When some smokers quit, they need to find something to do with their hands. You may want to pick up a new activity, such as knitting, writing or reading.

Some people find that they have a lot of extra time when they quit smoking, which can lead to boredom. Starting a new hobby is a good idea.

People who used to smoke during their breaks at work might need to find something new to do during those breaks after they quit smoking. Spending the break with non-smoking colleagues is a good option; taking a brief walk is also a healthy alternative.

Weekly Tip #11:

It's very common for people to experience withdrawal symptoms and cravings for several weeks after they quit smoking. Withdrawal symptoms are unpleasant but they'll pass. Cravings are momentary feelings and will pass within 20 minutes. When you experience withdrawal remind yourself that each symptom is a sign of recovery – your body is healing itself.

Weekly Tip #12:

Someone may offer you a cigarette while you are trying to quit smoking. This is a high-stakes situation because often one cigarette is enough to make you start smoking again. So, what can you do in these scenarios?

- you can politely say "No thanks" – no explanation required
- you can tell the person you've recently quit and ask for their support
- leave the scene momentarily (or avoid this person or situation in the future, if necessary)
